# Tau and Alpha Synuclein Synergistic Effect in Neurodegenerative Diseases: When the Periphery Is the Core

**DOI:** 10.3390/ijms21145030

**Published:** 2020-07-16

**Authors:** Elena Vacchi, Alain Kaelin-Lang, Giorgia Melli

**Affiliations:** 1 Laboratory for Biomedical Neurosciences, Neurocenter of Southern Switzerland, Ente Ospedaliero Cantonale, 6807 Lugano, Switzerland; elena.vacchi@eoc.ch (E.V.); alain.kaelin@eoc.ch (A.K.-L.); 2 Faculty of Biomedical Sciences, Università della Svizzera Italiana, 6900 Lugano, Switzerland; 3 Neurology Department, Neurocenter of Southern Switzerland, Ente Ospedaliero Cantonale, 6900 Lugano, Switzerland; 4 Department of Neurology, Inselspital, Bern University Hospital, University of Bern, 3010 Bern, Switzerland

**Keywords:** tau, Big tau, alpha-synuclein, intrinsically disordered proteins, peripheral nervous system, neurodegenerative disorders, proteinopathies, Alzheimer’s disease, Parkinson’s disease, axonal degeneration

## Abstract

In neuronal cells, tau is a microtubule-associated protein placed in axons and alpha synuclein is enriched at presynaptic terminals. They display a propensity to form pathologic aggregates, which are considered the underlying cause of Alzheimer’s and Parkinson’s diseases. Their functional impairment induces loss of axonal transport, synaptic and mitochondrial disarray, leading to a “dying back” pattern of degeneration, which starts at the periphery of cells. In addition, pathologic spreading of alpha-synuclein from the peripheral nervous system to the brain through anatomical connectivity has been demonstrated for Parkinson’s disease. Thus, examination of the extent and types of tau and alpha-synuclein in peripheral tissues and their relation to brain neurodegenerative diseases is of relevance since it may provide insights into patterns of protein aggregation and neurodegeneration. Moreover, peripheral nervous tissues are easily accessible in-vivo and can play a relevant role in the early diagnosis of these conditions. Up-to-date investigations of tau species in peripheral tissues are scant and have mainly been restricted to rodents, whereas, more evidence is available on alpha synuclein in peripheral tissues. Here we aim to review the literature on the functional role of tau and alpha synuclein in physiological conditions and disease at the axonal level, their distribution in peripheral tissues, and discuss possible commonalities/diversities as well as their interaction in proteinopathies.

## 1. Introduction

The most common human neurodegenerative affections are Alzheimer’s disease (AD) and Parkinson’s disease (PD), which respectively represent the first and second neurodegenerative disease in the elderly [1]. AD is primarily a cognitive disorder, characterized by the presence of abundant intraneuronal inclusions of assembled tau protein and extracellular amyloid plaques of Aβ peptides, and it is classified as a tauopathy. Whereas PD is prevalently a movement disorder, which is characterized by a single kind of intracellular amyloid assemblies, named Lewy inclusions, composed of alpha synuclein (αSyn) protein [2]. Most cases of these pathologies are sporadic, but a small percentage is inherited, caused by mutations in the genes encoding the proteins that make up the inclusions or the proteins that increase their production, underscoring the importance of inclusion formation for neurodegeneration. These proteins undergo a transformation from a soluble to an insoluble filamentous state, with a number of intermediates that are considered neurotoxic [3]. However, current knowledge still does not address the underlying early pathogenic mechanisms; therefore, AD and PD lacks an effective causal treatment and definitive early diagnostic biomarkers to be used in clinical trials for drugs discovery. In neurons, tau and αSyn share a significant role in cell trafficking and synaptic functions and both play a role in regulating mitochondrial homeostasis. Since the peripheral nervous system (PNS) encompasses axonal and synaptic compartments, where most likely the initial events in neurodegeneration happen, we aim to review the functional role of tau and αSyn in physiological conditions and disease, their distribution in PNS, and discuss possible commonalities/diversities as well as their interaction.

## 2. The Role of Tau in Physiological Condition in Axons

### 2.1. Structure of Tau

**Figure 1:**
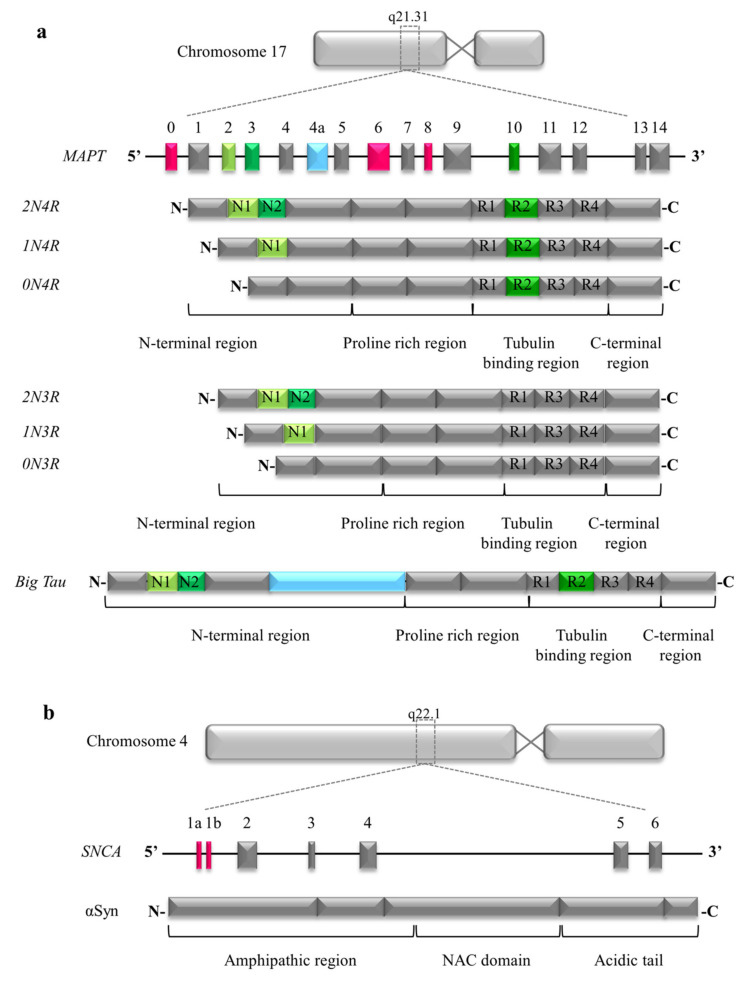
Gene, isoforms and functional domains of Tau and αSyn proteins. (**a**) Schematic representation of human *MAPT* gene present on chromosome 17. Its sixteen exons, through alternative splicing of exons 2, 3, and 10 (green), can generate 6 isoforms. Moreover, in peripheral tissue, the addition of exon 4a (light blue) generates the high molecular weight protein: Big Tau. The protein can be subdivided into 4 functional domains: N-terminal region, Proline rich region, Tubulin binding region, and C-terminal region. (**b**) Schematic representation of the *SNCA* gene present on chromosome 4. Exons 1a and 1b (red) are not translated. αSyn protein can be subdivided into 3 functional domains: amphipathic region, hydrophobic non-amyloid component (NAC) domain, and acidic tail.

Human tau is a microtubule binding protein coded by *MAPT* gene located on chromosome 17 (17q21.31) [4,5]. *MAPT* primary transcript consists of 16 exons, among which exons 1, 4, 5, 7, 9, 11, 12, and 13 are constitutively translated; whereas, exons 2, 3, and 10 are alternatively spliced [4]. Alternative splicing of exons 2 and 3 can include 0, 1, or 2 sequences of 29 amino acids, generating the isoforms 0N, 1N, and 2N respectively. Exon 3 never appears independently from exon 2 [6]. Alternative splicing of exon 10 can create isoforms containing 3 or 4 carboxy-terminal repeat domains, generating the isoforms 3R or 4R (Figure 1a).

Tau is a ubiquitous protein that is primarily expressed in the nervous system [7], with higher expression levels in neurons than astrocytes, oligodendrocytes, and Schwann cells [8,9,10]. In the brain tau, isoform expression is developmentally regulated: in fetal brain is expressed only the shortest isoform 0N3R [11], whereas the adult human brain shows all the isoforms [4]. In the adult brain, 0N and 1N isoforms represent 37 to 54% of total tau, while 2N isoforms are only 9% [12]. 3R and 4R isoforms are equally represented with few regionally differences: 4R isoform are more expressed in the globus pallidus, with a temporal frontal cortex distribution [13].

Tau protein can be divided into four domains: the N-terminal, the proline-rich domain, the microtubule binding region, and the C-terminal. The N-terminal is projected from the microtubule and interacts with other cellular elements. It can function as a spacer between microtubules and bridge between microtubules and the cytoskeleton or the plasma membrane, and together with the neurofilaments, it can determine the caliber of the axons [14]. The proline-rich domain conveys the link between tau and other proteins such as proto-oncogene tyrosine-protein kinase Fyn (Fyn) [15] and phospholipase C-γ (PLCγ) isozymes [16], involving tau in signal transduction pathways. The microtubule binding region allows the interaction between tau and microtubules and is involved in microtubule assembly, stabilization, and axonal transport. In particular, the tau isoforms 4R show greater affinity for microtubules compared to 3R tau isoforms [17,18]. Moreover, with the microtubule binding domain, tau can interact with other proteins such as F-actin [19] and αSyn [20]. Finally, less is known about C-terminal function [17]; however, this region is essential for the protein to fold and assume different conformations. Indeed, tau is considered an “intrinsically disordered protein” (IDP) and does not have a fixed secondary structure, but it is capable of multiple conformations [18,21]. Tau is highly dynamic in solution, although it features local secondary structure propensities, and an intricate network of transient long-range contacts related to the complexity of its functions and crucial for pathogenic aggregation, as demonstrated by nuclear magnetic resonance and cryo-electron microscopy studies [21,22]. Tau, when free in the cytoplasm, tends to acquire a “paperclip” conformation, in which N- and C-termini interact with the microtubule-binding repeat regions [18,21]. This conformation is modulated by post-translational modifications and interactions with microtubules or other proteins.

### 2.2. Big Tau in Peripheral Nervous System

In the PNS, tau transcript contains the additional exon 4a, generating the high molecular weight tau, named “Big tau” [23]. Big tau was described for the first time in 1984 by Drubin et al. [24]. The majority of the studies on Big tau were performed on mice and rats, whereas human data were obtained mainly by genomic analysis on transcript alignment [25]. In the PNS, Big tau expression is developmentally regulated and occurs postnatally [25]. It has been reported in sympathetic neurons [26], in cranial nerves, dorsal root ganglion (DRG) cells, and sciatic nerve [27,28]. However Big tau has been described also in neurons of the central nervous system (CNS) that extend axons into the periphery, i.e., in cerebellum, corpus callosum, and pyramidal tract [28]. Lower molecular weight isoforms are also expressed in PNS, in particular in sympathetic neurons [26,29], sciatic nerve, and trigeminal nerve [30]. Previous studies in rats suggest that Big tau can also have several variants due to the alternative incorporation of exon 6 that is never present in low molecular weight tau and leading to the detection of two isoforms of 110 and 90–95 kDa in the retina and optic nerve, not in DRG cells [28,31]. 

The incorporation of exon 4a carries the doubling of the N-terminal domain, and allows greater stabilization and spacing between the microtubules [32]. Long peripheral axons are subject to great shearing forces and Big tau may increase the structural stability. Moreover, it may contribute to the high rates of axonal transport observed in peripheral axons, thanks to the greater spacing between microtubules [33]. Comparing Big tau sequence from Xenopus and mammalian, only 22% of the exon 4a shows sequence identity compared to 75–83% for the microtubule-binding domain [34]. Plus, differently from the lower molecular isoforms, the insert has only two phosphorylated sites [25]. These observations suggest that the insertion of exon 4a serves mainly to increase the length of the N-terminal and not to allow the interaction of the protein with other cellular components [34]. Of interest due to the increased length of the N-terminus, it has been speculated that Big tau may have less propensity to conformational changes, aggregation, and spreading from neuron to neuron [35,36]. 

### 2.3. Tau and Axonal Transport

**Figure 2:**
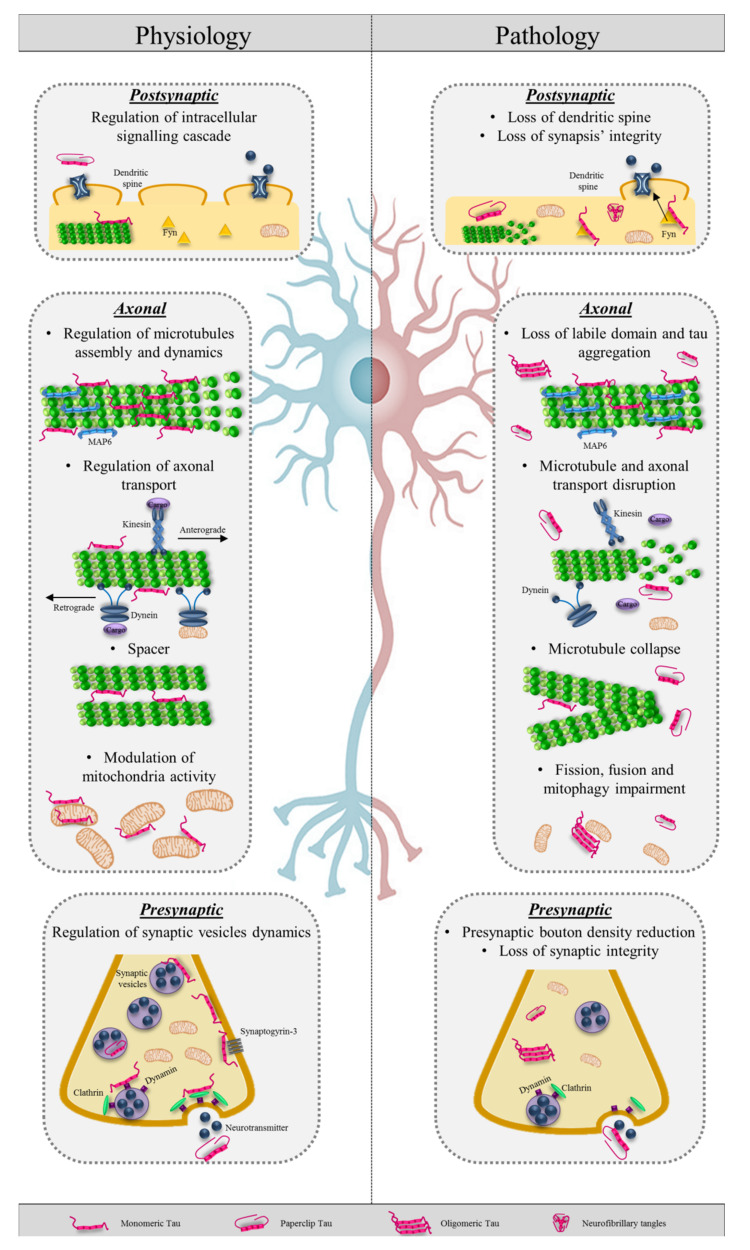
Tau protein in physiology and pathology. On the left, schematic representation of physiological tau functions at postsynaptic, axonal, and presynaptic level. On the right, pathological effects of abnormal (oligomeric and neurofibrillary tangles) tau in the three cellular compartments.

Although tau was traditionally considered a protein involved only in the regulation of tubulin polymerization and microtubule stability [37], it is well known that tau can fulfil multiple functions according to its different cellular locations (Figure 2). In healthy mature neurons, tau is mainly in the axons bound with the repeat-domain to microtubules, but it is also present in pre- and post-synaptic structures, in the nucleus, and associated with plasma membrane [38,39,40,41,42,43].

Along the axon, tau has a non-uniform distribution with a progressive proximal-to-distal increase concentration and with a higher amount near the growth cone [29]. In the distal axonal portion, tau promotes microtubule assembly in an unconventional way; indeed tau does not stabilize, but rather prevents genuine stabilizers, such as the microtubule associated protein 6 (MAP6), from approaching the microtubule labile domain, allowing the maintenance of a long labile domain and avoiding a slowdown in axonal growth [44]. The binding between tau and microtubules affects not only microtubule stability and growth but also influences axonal transport. Tau regulates the molecular motor proteins dynein and kinesin both directly and indirectly: (1) tau competes with dynein and kinesin for binding to microtubules, reducing the number of the motor proteins attached and also the binding frequency [45,46]; (2) tau influences the anterograde and retrograde mobility inducing the change of direction of dynein and the detaching of kinesin when a patch of tau is present along the microtubule [47]; (3) tau can activate the protein phosphatase 1 (PP1), which in turn activates the glycogen synthase kinase 3 beta (GSK3β) that induces the kinesin light chain cargo release [48]; (4) tau can bind to dynactin, favoring the link between dynein and microtubules [49]. Moreover, the N-terminal domain of tau, even if not directly bound to microtubules but projected away, can regulate microtubule dynamics, influencing the attachment and spacing between microtubules and other cell components [32]. The N-terminal works as a spatial separation, preventing microtubule overcrowding and favoring the transport of vesicles, mitochondria, and RNA along axons [50,51]. 

Finally, at axonal level, tau can act as a scaffolding protein: tau interacts with kinases and phosphatases such as PP1 [52], GSK3β [53], cyclin-dependent kinase 5 (Cdk5) [54], and Fyn [15], targeting these proteins to microtubules and facilitating their interaction with specific microtubule-associated protein substrates. Moreover, tau can directly interact with αSyn, which can modulate the phosphorylation of soluble axonal tau by GSK-3β [20] or by the protein kinase A (PKA) [55], influencing its link with microtubules.

### 2.4. Tau and Mitochondria

The presence of tau at the outer mitochondrial membrane and within the inner mitochondrial space has been recently demonstrated, suggesting that several physiological mitochondria-related functions may be regulated by tau [56]. Mutated tau inhibits mitochondrial transport toward the axonal tips, disrupting the energy supply, inducing oxidative stress, and leading to synapses and axon degeneration. Of interest, the clustering of mitochondria within the cell body induced by tau overexpression is influenced by tau isoforms; 4R tau has a stronger effect on mitochondrial localization than 3R isoforms, whereas no differences were observed between 0N, 1N, and 2N isoforms [57]. A direct co-localization between tau oligomers and mitochondria has been reported [58] and this interaction may increase the precipitation and phosphorylation of the pathological protein and it may alter mitochondrial quality control, leading to a redox imbalance [59]. Moreover, AD brains are characterized by mitochondrial fission and fusion dysregulation, shifting toward fission and resulting in abnormal free radicals’ production [60,61]. Tau accumulation also induces impairment of mitophagy [62], the physiological process that selectively degrades damaged mitochondria [63]. On the other hand, it has been shown that increased mitochondrial oxidative stress can induce tau hyper-phosphorylation. Indeed, in a mouse model lacking superoxide dismutase 2 and characterized by mitochondrial dysfunction and oxidative stress, a striking increase of tau phosphorylation at different epitopes was observed [64]. Mitochondrial impairment can be observed in AD brain at early stages and animal models for tau mutations and accumulation report alterations in mitochondrial function, trafficking [65] and mitophagy [66].

### 2.5. Tau at the Synaptic Terminals

The exact role of tau at the synaptic terminal is not fully unveiled: it appears to be involved in neuronal signaling and synaptic plasticity and integrity (Figure 2). These hypotheses arise from the fact that alterations in the tau protein alone lead to changes in these circuits. Indeed, tau mutation results in presynaptic bouton density and synaptic vesicle reduction and a decrease of postsynaptic dendritic spines in the hippocampus [67]. Moreover, tau reduction leads to synapsis’ integrity loss [39]. At the presynaptic level, tau can interact, both directly and indirectly, with proteins involved in synaptic vesicle dynamics, such as clathrin, dynamin, synaptophysin, synapsin 1, synaptotagmin, syntaxin-1B, αSyn, and β-synuclein [68,69]. Moreover, through its N-terminal domain, tau can bind to the synaptogyrin-3 and influence the release of synaptic vesicles [70]. At the postsynaptic level instead, tau seems to be involved in the regulation of intracellular signaling cascade through the binding with Fyn, regulating its localization and activity [71,72]. How tau can be present at the postsynaptic level and therefore in the dendrites is a question that has been tried to answer in recent years. Tau may be directly translated within dendrites [73], or tau may be released from pre-synaptic terminals and internalized into post-synaptic regions [74,75,76]. Finally, tau directly binds actin with the proline-rich domain and the microtubule binding domain, regulating its dynamics, polymerization, and stability [77,78]. Due to the crucial role of actin in synaptic remodeling, this interaction can also suggest a role of tau in synaptic function and, more in general, in the reorganization of the cytoskeleton network.

## 3. The Role of Alpha Synuclein in Physiological Condition in Axons

### 3.1. Structure of Alpha Synuclein

αSyn was initially identified in cholinergic vesicles of the Torpedo fish electric organ [79] suggesting from the beginning a functional role at the presynaptic level. It belongs to the super-family of synucleins, evolutionary highly conserved proteins, that also includes β and γ synuclein, sharing a common amino-terminal sequence, characterized by a different number of repeat regions [80]. αSyn is encoded by *SNCA* gene on the long arm of chromosome 4 (q22.1) and is characterized by a 140 amino acid (aa) sequence including three domains: the N-terminal region (1–60 aa), the non-amyloid component (NAC) region (61–95 aa), and the C-terminal (96–140 aa) (Figure 1b). The N-terminal region contains seven conserved repeat regions forming an amphipathic α-helix that is essential for membrane binding, and is stabilized by high-curvature membrane enriched of phospholipids like synaptic vesicles [81]; this region after acetylation can form α-helical oligomers [82] and of note it contains three sites of mutations causing familiar PD: A30P, A53T, and E46K [83]. The NAC region contains a hydrophobic motif that regulates oligomerization and fibril formation and is necessary and sufficient for aggregation of αSyn [84]. The C-terminus is polar, with the highest proportions of charged residues, and undergoes phosphorylation at multiple sites suggesting a mechanism of regulation; it is the least conserved among species and affects membrane binding [85]. In particular, phosphorylation at Ser129, nitration at Tyr125, Tyr133, and Tyr136 promotes oligomerization, conformational changes, and reduces membrane affinity [86]. In addition, the C terminal tail mediates αSyn interaction with other proteins [87].

αSyn is considered an IDP due to a high conformational plasticity according to environmental factors: in physiological condition, in human and rodent CNS, αSyn is an unfolded monomer but it can acquire a folded conformation or exists as a dynamic oligomer after interaction with other proteins or biological membranes [83,88].

### 3.2. Alpha Synuclein at the Synaptic Terminals

αSyn localizes specifically at the axonal terminal [89,90]; it displays a weak association with synaptic vesicles and is highly mobile at the presynaptic area, explaining its multitasking activity in cooperation with multiple proteins in regulating the synaptic machinery [85,91]. Of interest, it has been described that the specific brain region of zebra finch involved in bird song undergo a significant reduction in αSyn expression during song acquisition, suggesting a role for αSyn in synaptic plasticity [89].

**Figure 3:**
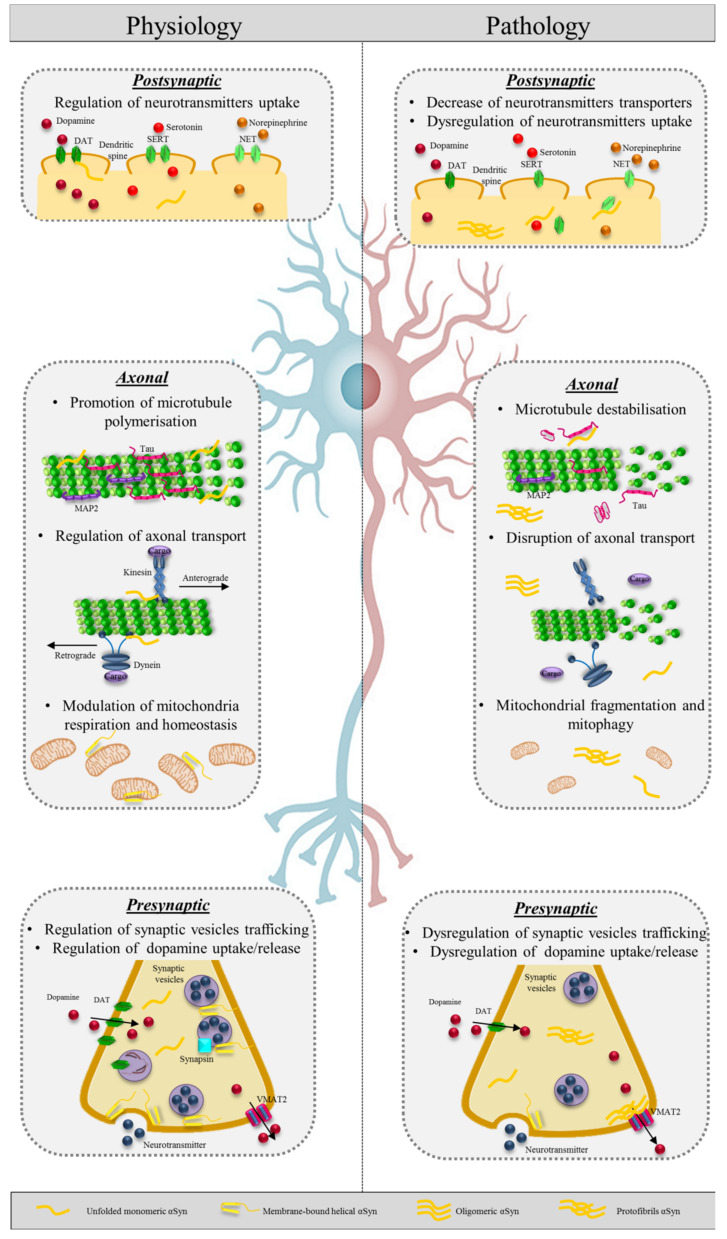
αSyn protein in physiology and pathology. On the left, schematic representation of physiological αSyn functions at the postsynaptic, axonal, and presynaptic level. On the right, the pathological effects of abnormal (oligomeric and protofibrillary) αSyn in the three cellular compartments. SERT:serotonin; NET: norepinephrine; DAT: dopamine transporter; VMAT2: vesicular monoamine transporter 2.

αSyn and β synuclein are specific inhibitors of phospholipase D2 (PLD2), which generates phosphatidic acid by hydrolysis of phosphatidylcholine and is localized at plasmatic membranes and vesicles; thus, synucleins are involved in synaptic membrane biogenesis and vesicle budding [92]. Since it interacts preferentially with small vesicles, αSyn likely regulates the mobility of synaptic vesicles between the recycling and resting pools [93] (Figure 3). In addition to its ability to bind lipid membranes, αSyn appears to interact with several proteins at synapsis like synphilin-1, which may act as an adaptor protein anchoring αSyn to proteins involved in vesicle transport and cytoskeletal functions [94]. On the surface of synaptic vesicles, αSyn interacts with a family of phosphoproteins called synapsins: in particular, it binds and cooperates with synapsin III to modulate dopamine release from nigrostriatal neurons [95] and a recent study shows that synapsin III knock out mice do not develop αSyn aggregates, nigrostriatal degeneration after overexpression of human wild type αSyn by adenovirus injection [96]. Moreover, αSyn interact with synaptic vesicle glycoprotein 2C (SV2C), cysteine string protein α (CSPα), and synaptobrevin/vesicle-associated membrane protein 2 (VAMP2) respectively, for vesicular function and soluble NSF attachment protein receptors (SNARE) complex assembly and function [97,98,99]. αSyn cooperates with Rab GTPs, a superfamily of numerous proteins that regulates axonal transport and synaptic vesicles trafficking; in particular, Rab3 and αSyn coordinate vesicles tethering at synaptic membranes [100], while Rab5 is involved in regulating the size of synaptic vesicles [101]. The interaction with Rab4A regulates protein sorting and transport, and αSyn is sorted to the early endosome by a mechanism dependent on Rab5A and to late endosome by Rab7 [102]. Interestingly, αSyn overexpression/aggregation can affect Rabs distribution and on the other hand Rabs dysregulation can influence αSyn pathology and propagation [103].

At synapsis, αSyn also regulates the neurotransmitter release rate, in particular of dopamine by interacting with vesicular monoamine transporter 2 (VMAT2), which is responsible for dopamine uptake [104]; and VMAT2 is found in Lewy bodies (LBs) [105]. Moreover, αSyn controls dopamine transporter (DAT) functions; in fact, in physiological conditions, it binds the C-terminus of DAT, increasing its levels at the plasma membrane and enhancing the uptake of extracellular dopamine [106]. In analogy, αSyn also controls the transporters of serotonin and norepinephrine [107].

A large number of studies support the hypothesis that synaptic dysfunctions are crucial players in retrograde degeneration of synucleinopathies [108,109].

The role of αSyn in neurotransmission at the synapses is well recognized, but αSyn also plays a crucial role in neurotransmitter synthesis, calcium homeostasis, mitochondrial function, and gene expression [110].

### 3.3. Alpha Synuclein and Mitochondria

αSyn directly modulates mitochondria by regulating their membrane potential, calcium homeostasis, cytochrome c release, and ATP production [110]. Despite αSyn lacking a canonical mitochondrial targeting sequence, the N-terminus domain of αSyn, which is rich in positively charged residues, mirrors the physical-chemical properties of mitochondrial targeting sequences and can adopt an α-helical conformation that can drive the anchoring of the protein to mitochondrial membranes [111,112]. The first N-terminal 32 amino acids have been proven to be fundamental for mitochondrial localization of the protein [111].

αSyn plays a role in physiological mitochondrial respiration: mice knockout for αSyn display decreased complex I/III activity, that likely derive from αSyn-mediated alterations of mitochondrial membrane lipid composition [113]. In fact, human fetal dopaminergic primary neurons exposed to αSyn gene silencing display an impaired connectivity between complex I and III and αSyn [114]. Finally, αSyn physiologically interacts with adenosine triphosphate (ATP) synthase [115]. In addition, dynamic processes such as mitochondrial fusion/fission and axonal transport are regulated by αSyn: in αSyn overexpressing dopaminergic cells, it has been shown that αSyn inhibits fusion and stimulates fission of mitochondria [116]. Mitochondrial fragmentation induced by overexpression of mutant αSyn (A53T, A30P, E46K) has been also observed [117]. In addition, the overexpression of αSyn in sensory neurons of living zebrafish embryos induced the fragmentation of mitochondria, leading to their swelling within the axon [118]. The mitochondrial pathology also includes mitochondrial DNA transport: mitochondrial motility was reduced by αSyn expression in SH-SY5Y cells and cultured neurons derived from human embryonic stem cells [119]. Further, αSyn transgenic mice display increased mitochondrial oxidative stress and DNA lesions [120].

### 3.4. Alpha Synuclein and Axonal Transport

Although αSyn is classically placed at synaptic terminals, it is now recognized that it also localizes at endosomes within axons [121] (Figure 3). It is in fact possible that in healthy neurons, αSyn selectively associates with phospholipidic membranes of different composition, including not only synaptic vesicles, but also endosomes and late endosomes [122]. It has been shown that αSyn associates with various cytoskeletal proteins for the homeostasis of cell structure and protein mobility [123]. αSyn interacts with α and β subunits of tubulin-promoting microtubules polymerization and enhancing the growth rate of axons [124]. αSyn co-localizes with dynein, which promotes retrograde transport and is a large motor complex composed of multiple subunits that require activation by dynactin; dynein dependent axonal transport is severely impaired without αSyn [125]. αSyn also plays a role for anterograde transport binding kinesin family member 5A (KIF5A), microtubule-associated protein 2 (MAP2), and tau [126]. αSyn interacts with 14-3-3 proteins [127]: the C-terminal phosphorylated form interacting with 14-3-3 modulates cytoskeletal and vesicular protein trafficking [128]. Moreover, 14-3-3 can be a αSyn chaperone and reduces its uptake and seeding properties [129]. αSyn also binds actin and modulates neurite outgrowth [130].

## 4. The Role of Tau in Neurodegenerative Diseases

Tauopathies are the most common proteinopathies of the human nervous system and are characterized by the deposition of abnormal tau protein, such as neurofibrillary tangles (NFTs) and neuropil threads (NTs) in nervous cells [17]. There are 27 different tauopathies, including AD, progressive supranuclear palsy (PSP), corticobasal degeneration (CBD), Pick’s disease (PiD), chronic traumatic encephalopathy (CTE), tangle-only dementia, fronto-temporal dementia with parkinsonism linked to chromosome 17 (FTDP-17), and argyrophilic grain disease (AGD) [131,132].

Tauopathies typically display at early phases a “dying back” pattern of neurodegeneration, with dystrophic axons, spheroids, axonal swellings, and evidence of disrupted fast axonal transport [133,134].

Several conformational variants of pathological tau, known as strains that can have dramatically different seeding and spreading abilities, have been reported [135]. For example, pathological tau isolated from brains of individuals with progressive supranuclear palsy (PSP) was conformationally distinct from and had greater seeding ability than pathological tau isolated from brains of individuals with AD [136].

### 4.1. Tau Mutations Impair Microtubules Binding Affinity

Abnormal tau protein can be the result of genetic mutations or post-translational modifications.

More than 80 *MAPT* mutations have been identified; they are mainly missense mutations, which alter tau sequence, or splicing mutations, which change the expression of the different isoforms. The majority of missense mutations affect the microtubule-binding domain, reducing tau affinity for microtubules and increasing tau tendency for aggregation [137,138]. Whereas, the majority of splicing mutations are within or near intron 10, increasing or reducing its inclusion in the transcript and thus changing the ratio between the 3R and 4R isoforms [139]. In AD, CTE, and tangle-only dementia, all six brain tau isoforms (3R and 4R) are present in neuronal cells as NFTs and NTs [17]. Instead in PSP, CBD, and AGD, only 4R tau isoforms are accumulated, and in PiD, only 3R tau isoforms are deposited as Pick’s bodies [17]. The 3R/4R imbalance is strictly linked to neurodegeneration and several mechanisms have been proposed to explain how this disequilibrium can lead to neuronal damage. 3R and 4R isoforms have a different affinity to microtubule [140], allowing a fine regulation of axonal transport. An increase in the 4R isoform, which has the strongest affinity to microtubules, lead to an higher probability of kinesin detachment [47]; whereas, an increase in the 3R isoform enhances the number of kinesin driving the cargo and the anterograde mobility [141]. The 3R/4R disequilibrium impacts the transport of cargos throughout neurons, damaging the cell. Moreover, due to the fact that the distribution and expression of 3R and 4R isoforms are crucial during brain development, a new hypothesis suggests the existence of a developmental origin of tauopathies. An imbalance in 3R/4R ratio may lead to genomic instability and aneuploidy, which has been observed in cells from AD patients and in animal models [142,143,144,145]. These aneuploidy cells in the adult human brain may present a higher vulnerability, leading to age-related neuronal degeneration [146].

Regarding the post-translational modifications, tau undergo phosphorylation [147], acetylation [139], ubiquitination [148], methylation [149], and truncation [139] which regulate its function within the cell. Also, in this case a dysregulation of these mechanisms can alter tau conformation and induce the detachment from microtubules. Moreover, only in tauopathies, tau undergoes also glycation, deamination, isomerization, and nitration [150].

The detachment of tau from the microtubules allows the exposition of two hexapeptide motifs, located in the second and third microtubule binding repeats, which display a high propensity to aggregate [21,151]. Tau dimerization occurs through interactions between the hexapeptide motifs of two different tau monomers [152]. Further, recruitment of tau leads to the formation of oligomers, which displays a β-structure [153,154,155]. Three strands forming a β-helix and 5 strands forming 2 antiparallel β-sheets regions together create a protofilament [156]. Protofilaments are packed symmetrically, arranged base to base, in paired helical filaments (PHFs) and asymmetrically, back to base, in straight filaments (SFs) [17,157]. PHFs and SFs can aggregate and generate NFTs in the neuronal body and NTs in neuronal process.

In the human brain, tau can be cleaved behind Thr123, generating an N-terminally truncated, tau124–441 fragment. This fragment displays a stronger affinity for microtubules than full-length tau, probably because the removal of the negatively charged N-terminal domain increases its binding to the negative surface of microtubules [158]. Several N-and C-terminally truncated Tau species are observed in AD and in other tauopathies [139]. It has been shown that truncation of tau could generate tau fragments with a higher tendency for aggregation, probably owing to the disruption of the paperclip structure of tau [159].

### 4.2. Pathological Tau Hampers Axonal Transport

The majority of tau mutations determine a conformational change, the detachment of the protein from the microtubules, and the possibility of tau to create aggregates (Figure 2). This can lead to both the loss of tau physiological functions and acquisition of new abilities that can influence the normal cellular physiology.

The delocalization of abnormal tau from the microtubules to the cytoplasm prevents the microtubules assembly and stabilization and the formation of an organized cytoskeletal network [160,161]. The detachment results in microtubule density and axonal caliber alterations [131] and prevents tau from playing its role as a scaffolding protein. Mislocalized tau and compromised microtubules induce axonal transport impairment. Abnormal tau inhibits the access of kinesin to microtubules track and reduces binding frequency and mobility of dynein and kinesin [162]. Moreover, detached tau exposes the phosphatase-activating domain (PAD), generally sequestered in physiological tau [163,164], which can activate in a constitutive and unregulated manner the PP1/GSK3β pathway, inducing a progressive kinesin light chain cargo release [48,165]. Axonal transport disruption means incorrect transport of proteins, vesicles, mitochondria, and peroxisomes [166]. An incorrect relocation of the mitochondria in peripheral axon, a decrease in ATP synthesis, glucose and lipid metabolism, and an increase in oxidative stress lead to progressive dying-back degeneration [167]. Moreover, abnormal tau can increase intracellular Ca^2+^ levels by directly inhibiting plasma membrane Ca^2+^ ATPase [168] and by reducing axonal mitochondria [169]. The increase of Ca^2+^ in the neuron promotes the abnormal activation of calcium-activated proteases and the proteolysis of critical cytoskeletal protein components [170]. At the synaptic level, the increase of Ca^2+^ caused by abnormal tau leads to an inhibition of synaptic vesicle exocytosis and synaptic transmission [171]. Moreover, in a mouse model, it has been observed that oligomeric tau can completely deregulate synaptic activity, inducing aberrant long-term potentiation/depression [67,172]. Finally, the impossibility of maintaining the neuronal shape and the loss of contacts with neighboring cells, through synaptic afferents and efferents, induces synapses deterioration [173].

### 4.3. Neuroanatomical Stages of Tau Accumulation in Alzheimer’s Disease

In an AD brain, pathological hyperphosphorylated tau aggregates appear first in the axons of locus coeruleus of the pontine tegmentum nerve cells (subcortical stage 1a) and then in the somatodendritic compartment (subcortical stage 1b) [174]. The migration from the axon to the somatodendritic compartment could be caused by defective mechanisms in functionally impaired axons [175]. Indeed, during AD pathogenesis, hyperphosphorylated tau is free in the cytoplasm of both axon and somatodendritic area. Over time, hyperphosphorylated tau becomes less soluble and starts to aggregate and to be confined solely to the somatodendritic compartment, where pre-fibrillar structures develop [175]. In the axon instead, hyperphosphorylated tau remains for a longer period in a non-fibrillary state and can be transferred into presynaptic terminals, where they become available for synaptic transport [176]. From the locus coeruleus, pretangle tau reach the transentorhinal region of the cerebral cortex, where it is possible to observe the first NFTs [174]. Subsequently, neurofibrillary lesions spread from the transentorhinal region into the olfactory bulb (NFT stage I); the entorhinal region and the hippocampal formation (NFT stage II); basal temporal neocortex (NFT stage III); the temporal, insular, and frontal neocortex (NFT stage IV). Finally, in the last two stages, NFTs also invade the neocortical association areas (NFT stage V and VI) [174]. How the pathology moves from an area to another is still a cause for debate. Several hypothesis have been proposed: (1) locus coeruleus neurons send aberrant signals to the cortical nerve cells of the transentorhinal region that might induce a transient overproduction of tau; (2) axonal hyperphosphorylated tau is carried by synaptic vesicles and conveyed to the transentorhinal neurons post synapsis; (3) tau spread in a “prion-like” manner [174]. In the transentorhinal region, pretangle tau develops to NFT.

Despite the accumulation of abnormal tau being the basis of all tauopathies, these diseases differ from each other with regard to the brain areas affected, the kind of tau aggregates, and the cell types in which the aggregates are found, which is either neurons or astrocyte or oligodendrocytes. For example in PSP, different from AD, pathological tau accumulations start in the neurons of subcortical and brainstem nuclei, in the oligodendrocytes of the globus pallidus, and in the astrocytes of the striatum, followed by tau accumulation in cortical astrocytes, neurons, and oligodendrocyte, respectively, with a fronto-parietal to temporal to occipital sequence [177].

## 5. The Role of Alpha Synuclein in Neurodegenerative Diseases

αSyn forms misfolded aggregates in a group of neurodegenerative diseases collectively known as synucleinopathies, including PD, dementia with Lewy bodies (DLB), and multiple system atrophy (MSA) [178]. PD and DLB are thought to be a spectrum of disorders characterized by intraneuronal aggregates called LBs and Lewy neurites (LNs), whereas MSA is characterized by abundant oligodendroglial αSyn aggregates, termed glial cytoplasmic inclusions (GCI), with rare neuronal inclusions [179].

The central role of αSyn in PD is extensively supported by neuropathological and genetic studies: the presence of intracellular inclusions such as LBs and LNs that are principally constituted by aggregated αSyn [180] and the discovery of familial PD caused by mutations in the *SNCA* gene (A53T) [181]. Since then, several other point mutations of *SNCA* (A30P, E46K, H50Q, G51D and A53E) have been discovered to cause PD and DLB [182,183,184,185,186,187]. Plus, duplication and triplication of αSyn locus have been described [188,189], demonstrating a gene dosage effect with earlier onset and faster disease progression in subjects with triplication compared to those with duplication [188]. Besides *SNCA*, more than 20 PD relate genes have been identified, encompassing *LRRK2*, *GBA1*, *PINK1*, *PARK7*, and *PARK2* [190,191,192]. Not only sporadic but also genetic PD display accumulation of brain αSyn inclusions, and genetic mutations are mostly involved in αSyn aggregation or clearance pathways [193]. αSyn inclusions are thought to spread along neuronal connections in a stereotypical pattern in the nervous system and pathological forms of αSyn propagate in cell culture models and in vivo in a prion-like manner. Moreover, different conformational polymorphs of αSyn, called strains, exhibit distinct biochemical, physical, and structural features and this had led to the view that the clinical heterogeneity observed in synucleinopathies might be due to distinct pathological αSyn strains [194].

### 5.1. Axonal Transport Dysfunction Caused by Alpha Synuclein Aggregates May Be the Early Event in Neurodegeneration

There is a large amount of evidence that defects in axonal transport, due to aggregated αSyn, cause neuronal dysfunction and are associated with the early phase of PD [195]. The nigrostriatal system in PD shows a “dying-back“ pattern of degeneration, which starts at the axonal terminals and proceeds centripetally to the soma [196]. This pattern of axonal damage resembles Wallerian degeneration that has been widely studied for lesions of peripheral nerves and is characterized by early microtubules fragmentation, growth cone collapse, and axonal retraction [197,198]. Indeed, αSyn oligomers disrupt axonal integrity in induced pluripotent stem cells derived human neurons by altering the association with kinesin, which is essential for the axonal transport [126]. Further proteomics analysis of LBs showed enrichment of dynein, dynactin [199], and tubulin, which seems to potentiate αSyn fibrillation [124]. Moreover, the principle mutations linked to PD are in genes encoding for proteins related to the microtubules: leucine rich repeat kinase (LRRK2), parkin, and αSyn. LRRK2 modulates microtubule acetylation and organization [200] and mutated LRRK2 inhibits axonal transport of mitochondria by binding preferentially to deacetylated microtubules [201]. PARK2 mutations lead to the production of abnormal parkin that induces destabilization of microtubules in murine [202] and human dopaminergic neurons [203]. Concerning αSyn, it is known that it interacts with multiples cytoskeletal proteins contributing to the homeostasis of cell structure and protein mobility (Figure 3). In fact, αSyn acts as a microtubule dynamase, which regulates microtubule nucleation and catastrophes at the growth cone, and αSyn mutations induce tubulin aggregation [204]. The fact that the majority of point mutations in the *SNCA* gene map to the putative tubulin-binding site [200] underlines the importance of this pathway in causing the pathology. Importantly, in genome-wide association studies, one of the genetic regions most significantly linked to sporadic PD is the haplotype 1 (H1) located on chromosome 17q21, which associates also to atypical parkinsonisms, i.e., PSP and CBD. The gene that mostly associates with PD in H1 haplotype is *MAPT*; the impact of *MAPT* variants on axonal trafficking is high and further underlines the crucial role of axonal transport in PD. Indeed, LBs and LNs are preferentially formed in projection neurons with very long, thin unmyelinated axons [205] that are more susceptible to axonal transport deficit, energetic/metabolic stress, mitochondrial failure, and lack the trophic support of glial cells, such as nigrostriatal dopaminergic neurons, cardiac and skin sympathetic neurons, and gastro-enteric autonomic systems. Indeed, autonomic dysfunction symptoms and cardiac noradrenergic nerves’ impairment, as demonstrated by radio-labeled meta-iodobenzylguanidine (MIBG), are early features in the course of PD [206]. Moreover, pathological evidence of aggregated αSyn has been demonstrated in vivo and post-mortem in gastro-intestinal tissue, skin biopsy, and submandibular glands (see the paragraph “Alpha synuclein in the peripheral nervous system as a biomarker for synucleinopathies”). In addition, nigrostriatal neurons are characterized by extensive branching that exponentially increases the number of synapses at each axonal terminal, determining a substantial rise of the expression of αSyn [207]. The expression level of substrates, amplifying the pathogenic seed, could contribute to the selected vulnerability of different neuronal populations according to the recent theories on the misfolded protein transmission in neurodegenerative diseases [208,209].

### 5.2. Neuroanatomical Stages of Alpha Synuclein Accumulation in Parkinson’s Disease

In PD brain, αSyn aggregates appear first in the olfactory bulb and in the dorsal motor nucleus of the vagal nerve (DMV) [174,210]. In the viscemotor neurons of DMV, it is possible to observe both spindle-shaped and globular somatic inclusions, which are respectively LNs and LBs. Whereas, LNs are prevalently present in the unmyelinated and long axons of the projection cells of DMV in the medulla [174,210]. In stage 2, retrograde axonal and trans-neuronal transport via descending fibers drive Lewy pathology from the nucleus of the vagal nerve to the lower raphe nuclei, magnocellular portions of the reticular formation, and the locus coeruleus [175,210]. During stage 2 and 3, neurons in the spinal cord, targets of the lower brainstem nuclei projections, develop Lewy pathology. In particular, neuronal cells of the intermedio-lateral nucleus in layer 7 are affected in stage 2, while the large nociceptive projection neurons in layer 1 and the motoneurons in layer 9 are affected in stage 3 [174,211]. In stage 3, Lewy pathology also diffuses into the mesencephalic tegmentum and basal portions of the prosencephalon. At stage 4, LBs and LNs are found in the forebrain, in particular in the transentorhinal region in the anteromedial temporal lobe. At this point, aggregated αSyn gradually spreads throughout the entire neocortex: in high-order sensory association areas and prefrontal fields of the neocortex, such as subgenual, insular and anterior cingulate areas (stage 5) and in the first-order sensory association areas, primary sensory and premotor/motor fields (stage 6) [175,210].

## 6. Pathological Tau and the Peripheral Nervous System

Tau pathology preferentially affects neurons with long, scarcely myelinated, axonal projections in CNS; in fact, in AD subjects, neurons of the neocortex, entorhinal cortex, and hippocampus are the most affected [212,213,214,215]. Although PNS encompasses neurons with long axons, relying on axonal transport for energetic-metabolic supply, only a limited number of studies investigate pathological tau in periphery.

Schwann cells provide mechanical and trophic support to peripheral axons and are essential for the maintenance of peripheral nerves’ physiological function. It has been reported that tau is expressed in Schwann cells and is involved in Schwann cells’ mobility and phagocytosis ability; indeed, Yi and colleagues demonstrate that in *MAPT*-knockout mice, the decrease of tau expression reduced Schwann cell migration and suppressed the ability to clear debris after sciatic nerve injury [10]. Thus, tau pathology can induce neurotoxicity affecting not only neurons, but also damaging Schwann cells and preventing their trophic and supportive role. Iper-phosphorylated tau has been observed in cutaneous Schwann cells [216].

### 6.1. Tau and Autonomic Nervous System

The presence of physiological tau in the myenteric (Auerbach’s) and submucosal (Meissner’s) plexuses has been demonstrated in axons, cell bodies, and dendrite-like structures [217,218,219,220]. In adult human enteric plexus, mainly two isoforms of tau (1N3R and 0N4R) are expressed [219,221], while the detection of Big tau is controversial in the literature—some authors were unable to detect it and others showed a band of approximately 110 kDa by Western blot [219,221]. The presence of phosphorylated tau has also been investigated. Lionnet et al. demonstrate that tau is phosphorylated at Serine 202, Threonine 205, Serine 396, and Serine 404 in human colon specimen of both healthy and PSP subjects and this phosphorylation is resistant to lambda phosphatase treatment [219]. They also observed C-terminal truncated tau with an antibody specific for tau cleaved at Asparagine 421, but again no differences were observed between healthy and PSP subjects [219]. In addition to these modifications, Dugger and colleagues observed phosphorylation at Threonine 231 in AD and healthy subjects [221] and a threadlike immunoreactivity within the muscularis and ganglion cells of the submucosa and myenteric plexus of AD patients [217]. NFTs were observed in the celiac, stellatum, and sympathetic paravertebral ganglia of elderly subjects with or without tauopathies [222,223], suggesting that the occurrence and frequency of NFTs in the sympathetic ganglia may be related to aging and not to the presence of brain proteinopathies. In submandibular glands of patients with AD, PSP, and CBD, tau has been observed phosphorylated at Serine 202, Threonine 205, and Threonine 231 in stromal nerve fascicles, ganglion cells, and threadlike elements [217].

### 6.2. Tau and Somatosensory Nervous System

The content and phosphorylation state of tau in the somatosensory fibers, in particular in the sciatic nerve, has been described during ageing and in AD patients [224]. In the sciatic nerve of elderly subjects, both Big tau and lower molecular weight isoforms are present. Tau phosphorylation increases with age. In AD subjects instead, the amount of physiological tau decrease over time, while the phosphorylation state increases, even though no aggregated tau is observed [224], suggesting that the increased phosphorylation of tau in peripheral neurons may not necessarily be accompanied by aggregated formation. However, these studies on PNS are relatively few, with small sample sizes in each diagnostic group, and may have been confounded by the limited sensitivity and specificity of reagents available at the time. More recently phosphorylated tau has been described in cervical skin peripheral nerve terminals of patients with PSP [216].

### 6.3. Tau and Olfactory Nervous System

Olfactory receptor neurons are bipolar cells present in the epithelial lining of the nose [225]. Their axons project to the olfactory bulb, forming the olfactory nerve that provides information to the CNS. Physiological tau is present especially in the olfactory nerve and olfactory bulbs, with a lower expression in the receptor neurons and in axonal bundles of the olfactory epithelium submucosa [226]. Apparently, Big tau is not expressed in the bipolar cells of olfactory ganglia and olfactory nerve [33]. Olfactory dysfunction is an early and common symptom in several neurodegenerative diseases, including AD, CBD, frontotemporal dementia, to a lesser extent in PSP, as well as in PD and synucleinopathies [227]. In AD patients, olfactory symptoms correlate with the early phases [228] and advancement of the AD pathology [229,230] and olfactory dysfunctions reflect neurodegenerative disease pathology in the brain [230]. AD olfactory epithelium shows a striking accumulation of dystrophic neurites [231,232]: abnormal neuronal processes characterized by aberrant sprouting, dystrophic expansion, and proteins accumulation, among which, physiological and abnormal/aggregate tau and αSyn [233]. These neuritic abnormalities are not specific and exclusive for AD patients, due to their presence also in healthy subjects and patients with other neurodegenerative diseases [234]. Nevertheless, in AD olfactory epithelium PHFs pathological lesions are significantly more frequent and more abundant compared to normal elderly controls or patients with other neurodegenerative diseases [233]. Abnormal tau was described in dystrophic neurites coursing through the lamina propria and in the neuron somata with a morphological appearance typical of NFTs [233]. Moreover, these lesions correlate with higher levels of PHF tau pathology in the cortex of AD subjects [233]. Finally, NFTs have been observed also in dendrites of the olfactory receptor cells and in nerve bundles [235]. To date, the olfactory mucosa seems to be the peripheral tissue with a major diagnostic utility for AD, even with some limitations in terms of specificity.

## 7. Pathological Alpha Synuclein and the Peripheral Nervous System

### 7.1. Dual Hit Hypothesis

In 2007, Braak proposed the dual-hit hypothesis on the origin of PD. Since αSyn aggregates appear first in the olfactory bulb and in the DMV, it was postulated that PD possibly originates also from the PNS gastro-enteric synapses and it invades the CNS via retrograde axonal transport [236]. This hypothesis has been supported by many clinical observations and experimental settings. αSyn can be detected in the gut of PD patients up to 20 years before the diagnosis [237] and truncal vagotomy appeared to lower the risk of developing PD of 40–50% after 10–20 years [238]. In a transgenic rat model with excess levels of αSyn, the injection of αSyn fibrils into the duodenum fully recapitulated the trans-synaptic propagation through the vagus nerve to DMV, through the sympathetic connectome to the celiac ganglion and IML, and then rostrally to the brainstem with involvement of locus ceruleus and substantia nigra. This model also provided the first evidence of a secondary anterograde transport to the stomach and heart [239].

However, due to the high clinical heterogeneity of PD patients and the fact that a small number of PD subjects do not present pathology in the DMV, it has been proposed more recently that PD can be divided in PNS-first and CNS-first types. The PNS-first phenotype is associated with REM sleep behavior disorder (RBD) during the prodromal phase and displays a marked autonomic damage before the dopaminergic system involvement, while the CNS-first is RBD negative during the prodromal phase and presents a marked involvement of the nigrostriatal system before the autonomic PNS damage [240].

### 7.2. Alpha Synuclein in the Peripheral Nervous System as a Biomarker for Synucleinopathies

The involvement of PNS, either early or later on in the course of the disease, has been widely demonstrated in PD and synucleinopathies. In fact, in PD and DLB, αSyn aggregates have been detected throughout the PNS in sympathetic ganglia, enteric nervous system, cardiac and pelvic plexus, submandibular glands, adrenal medulla, and skin [241]. In MSA, αSyn aggregates have been demonstrated in sympathetic ganglia [242], in Schwann cells cytoplasm [243] and in skin autonomic nerves [244]; of note, a study on sural biopsy showed a reduction of small unmyelinated fibers (somatosensory and autonomic) in 23% of MSA cases [245] and a mild degeneration of cardiac sympathetic nerves has been described [206]. These findings have important implications for the discovery of in vivo biomarkers of disease that are so urgently needed for neurodegenerative diseases. A recent systematic review and meta-analysis on determining the most suitable tissue for assessing αSyn deposits in PD found that skin biopsy using anti-phosphorylated αSyn antibodies displayed the best diagnostic accuracy, when compared to gastro-intestinal tract, submandibular glands, minor salivary glands, parotid glands, and olfactory epithelium [246]. Indeed, phosphorylated αSyn has been found in skin small fibers nerves innervating autonomic structures [247] in PD, and of note also in RBD patients, suggesting the potential value of skin biopsy as an early biomarker of disease [248,249]. More recently, aggregated αSyn has been demonstrated in skin nerves by conformational antibodies, recognizing oligomeric forms of the protein [244,250] and by using proximity ligation assay technology in the skin [251]. In addition, skin biopsy offers the great advantages of being minimally invasive, compared to other biopsy sites like the gastro-enteric system; it can be repeated in time during follow-ups and offers the unique opportunity to access the sympathetic structures in the skin (sweat glands, small arterioles, muscle arrector pili), which animal models have demonstrated as possibly involved early [239]. Of great interest, skin biopsy can be used to detect small fiber neuropathy. Several clinical studies have shown that PD patients develop a small fiber neuropathy [252] which is most likely expression of the neurodegenerative process in itself and seems to correlate to disease progression [244,253]. These findings imply a possible role of small fiber neuropathy as a biomarker of disease.

## 8. Tau and Alpha Synuclein Cross Talking in Causing Neurodegeneration

Among neurodegenerative diseases, pure single proteinopathies are rare: they generally exhibit associated deposits of other misfolded aggregated proteins. For example, co-occurrence of tau and αSyn inclusions in brain pathologies is frequent: more than 50% of AD patients show LBs [254], while tau pathology has been observed in sporadic and genetic PD [255,256]. Moreover, tau and αSyn co-localization has been described in both LBs and NFTs [257,258], suggesting that the interaction between tau and αSyn may influence the development and spreading of neurodegeneration. Of note, genome-wide association studies have shown that genetic variants of *MAPT* mutations were associated with both tauopathies and synucleinopathies [259,260], while *SNCA* mutations were associated with NFTs’ pathology [261].

Tau and αSyn are present at both axonal and synaptic levels and can physically interact: the C-terminus of αSyn links the microtubule-binding region of tau [20]. This interaction influences the binding of tau to microtubules contributing to the regulation of microtubules’ stability and polymerization. Modifications in these regions may influence their interaction; for example, phosphorylation of the serine 214 residue of tau was identified to increase αSyn binding [55]. Moreover, αSyn can influence tau functions, also regulating its phosphorylation by GSK-3β and PKA interaction. Due to the fact that oxidative stress can stimulate αSyn, in pathological conditions, αSyn may lead to excessive phosphorylation of tau by GSK-3β [262]. Furthermore, tau and αSyn also promote each-other’s aggregation in in-vitro experiments: αSyn’s NAC domain can induce tau polymerization and tau promotes formation of αSyn inclusions [256,263].

It has been shown that αSyn disrupts the actin network, causing dynamin related protein 1 (Drp1) dependent mitochondrial fission defects [264] and a very similar mechanism underlines tau pathology in mitochondria [265], implying that αSyn and tau toxicity converge onto a common knot that is f-actin stabilization and mitochondria damage [266].

Indeed, transgenic mouse models with the co-occurrence of tauopathy and synucleinopathy display both protein aggregations, cognitive and motor deficits, further suggesting a synergic effect of the two pathological proteins on neurodegeneration. Indeed, mutant P301L tau rats display a higher level of αSyn and its phosphorylated form and develop motor dysfunction [267,268] and mice overexpressing K396I tau show L-Dopa sensitive parkinsonism [269]. In analogy, animal models of synucleinopathies show cognitive deficit and abnormal tau accumulation, like in the case of mice overexpressing A53T and E46K αSyn, who display abundant tau accumulation [270,271].

## 9. Tau and Alpha Synuclein Commonalities and Diversities

Normal and pathological tau and αSyn share biological and biophysical properties: they have a high content of charged amino acid residues and are highly soluble in aqueous buffers [272]. Moreover, they have long half-lives in vivo and as ‘natively unfolded’, they are heat-stable [273]. They also contain stretches of hydrophobic residues that are involved in forming the core of the assembled fibrils [274]. Further, both proteins are phosphorylated, and these modifications have been implicated in facilitating their conversion into fibrils [275]. αSyn and tau fibrils, either assembled in vitro from recombinant protein or derived from pathological aggregates, share common properties with amyloid: they form 10–15 nm diameter filaments that are detected by Congo red and Thioflavin S [276,277].

From a functional point of view, tau and αSyn share a significant role in cell trafficking and synaptic functions at the axonal level and both play a role in regulating mitochondrial homeostasis. In fact, pathologic αSyn and tau cause mitochondrial dysfunction and oxidative stress, and tau aggregates amplifies the neurotoxic effects on mitochondria by interacting with αSyn; on the other hand, oxidative stress caused by mitochondrial impairment induces aggregation of both αSyn and tau [278].

### 9.1. IDPs and Neurodegenerative Diseases

Similar to most of the proteins involved in neurodegenerative proteinopathies, tau and αSyn are IDPs [87,279,280]. In fact, tau and αSyn are intrinsically disordered hubs with highly extended proteomes [281]. IDPs have been shown to have redundant biological functions, including cell signaling, transcription, translation, and cellular trafficking/ synaptic function [87,279,280]. Indeed, αSyn displays a high predicted disordered sequence (90%) and an elevated binding promiscuity with 416 interactors; similarly, tau has 77.6% of predicted disorder and 73 binding partners [281]. IDPs exert their molecular functions using conformational flexibility and heterogeneity and are tightly regulated by extensive post-translational modifications such as phosphorylation, acetylation, and glycosylation [281]. The binding selectivity of charged IDPs like αSyn and tau may be also determined by regulatory mechanisms that are dependent on its subcellular localization, or during relevant stages of development or cell cycle [83]. These properties at the same time can be responsible for the different conformational species that these proteins can generate in different cellular milieus, causing different toxicity and spreading patterns characterizing the multiple clinical and neuropathological phenotypes of neurodegenerative diseases.

### 9.2. Aggregation, Propagation, and Prion Concept

Tau and αSyn are soluble proteins showing a propensity to aggregate in insoluble filaments, which constitute the end point of the aggregation process that is localized intracellularly in neurons and glia cells, differently from β-amyloid that accumulates in the extracellular space. The process involves the formation of a pathological seed, a rare and energetically disadvantageous event, which requires high protein concentration and exposure of amide groups. Pathological seeds induce the rapid assembly of other soluble protein monomers, and then the subsequent fragmentation generates new seeds with an acceleration of the process [2]. In analogy to prions, misfolded proteins generate seeds that can guide the aggregation of further homologous protein and propagate transcellularly. The event is considered non-cell autonomous and propagate to distant regions of the brain in a stereotypical manner for both tau and αSyn, predicted by the pathological staging of tauopathies and PD [3].

Of interest, recently developed ultrasensitive technologies based on the aggregation properties of different pathologic seed of tau and αSyn, like Real Time Quaking Induced Conversion assay (RT-QuIC), developed in the field of prion diseases, and Protein misfolding cyclic amplification (PMCA), are promising tools in detecting pathological aggregates in CSF, brain and olfactory mucosa homogenates for the diagnosis of several synucleinopathies and tauopathies [282,283,284].

### 9.3. Pathological Tau and Alpha Synuclein Target Different Neuronal Cells and Display Different Spreading Patterns

In contrast to tau that is distributed throughout the neuronal cells, αSyn localizes specifically at the synapses, with relatively low presence in the cell bodies, dendrites, or in extra synaptic locations along axons [89,90]. In adult neurons, αSyn and tau may interact with each other, when low levels of αSyn are expressed in the cell body/axon before being transported to synaptic terminals. However, it is especially under pathological conditions, when impaired axonal transport leads to accumulation of αSyn along axons and cell bodies, that this interaction becomes more likely [274].

Pathological aggregates of both proteins involve mainly projection neurons, with long and sparsely myelinated axons, but the pathological cascade starts at different preferential sites according to the disease, locus coeruleus in AD, and olfactory bulb/DMV in PD; and propagate in a standardized pattern. Further, AD and tauopathies display pathological protein inclusions that remain confined mainly inside the CNS, while in PD, they also involve the PNS autonomic and somatosensory nervous systems [174]. Besides, multiple evidences and experimental studies have demonstrated the αSyn transmission from the PNS to CNS, while the evidence of tau seeds spreading from the periphery to the brain is not as strong as for αSyn. Indeed, the apparent minor impact of tauopathies on PNS compared to synucleinopathies could be due to the less “pathogenic” phenotype of Big tau in peripheral nervous tissues with a possible minor propensity to misfolding, aggregation, and spreading. More studies on peripheral Big tau in humans are certainly needed to confirm this hypothesis and to investigate the intriguing possibility of an “innate” mechanism of neuroprotection exerted by Big tau [25].

## 10. Conclusions

Synucleinopathies and tauopathies are characterized by the accumulation of misfolded proteins in selected populations of neurons: whether this pathological accumulation is the cause or the effect of axonal transport dysfunction is still debated. The identification of familiar forms of disease caused in the majority of cases by genes encoding for proteins involved in axonal transport and the evidence of toxic models of disease caused by chemical agents causing axonal transport deficits and neurodegeneration support the theory that initial axonal transport impairment can trigger neurodegeneration. Another key player is mitochondrial dysfunction, which is apparently strictly linked to axonal transport derangement and is a common feature in synucleinopathies and tauopathies, both defined by a dying-back degeneration pattern. In addition, myelination could have a role, since a striking susceptibility to misfolded protein pathology is shared by small diameter and unmyelinated or sparsely myelinated nerve fibers. An explanation could certainly be the lack of trophic support by myelinating glial cells but it is of interest that myelin and/or myelinating cells also influence the density of axonal mitochondria, such that unmyelinated axons display a higher density of mitochondria than myelinated internodes [285,286]. Plus myelin also locally increases axonal calibre by modulating axonal neurofilament transport [287,288], and phosphorylation/spacing [289].

Another recent and important concept is that tau and αSyn are IDPs generating different conformational strains, which are likely responsible for the heterogeneity of pathological findings and clinical phenotypes [209]. Several common routes of pathogenesis underlying IDP-related diseases and protein misfolding diseases suggest promising potential points of intervention to prevent neurodegeneration.

In conclusion, more attention to axonal biology can add important clues to the pathogenesis of neurodegenerative disease and shed light on possible novel neuroprotective pathways. The cell death paradigm of neurodegeneration will need to be substituted or at least associated with the axonal degeneration paradigm as an early crucial event in the neurodegenerative pathways causing misfolded proteins-derived pathologies.

Furtherly, PNS deserves more attention because it encompasses axonal and synaptic compartments, where the initial events in neurodegeneration most likely happen. In addition, due to the easy accessibility of PNS tissues, they offer the great opportunity to detect potential novel biomarkers of disease that are urgently needed for neurodegenerative conditions. The peripheral nervous tissues represent an easily accessible window to brain pathology in neurodegenerative diseases.

## Abbreviations

AD	Alzheimer’s Disease
PD	Parkinson’s Disease
αSyn	Alpha Synuclein
PNS	Peripheral Nervous System
CNS	Central Nervous System
IDP	Intrinsically Disordered Proteins
aa	amino acid
NAC	non-amyloid component
NFTs	NeuroFibrillary Tangles
NTs	Neuropil Threads
PSP	Progressive Sopranuclear Palsy
CBD	CorticoBasal Degeneration
PiD	Pick’s Disease
CTE	Chronic Traumatic Encephalopathy
AGD	Argyrophilic Grain Disease
PHFs	Paired Helical Filaments
DMV	Dorsal Motor Nucleus of the Vagal nerve
DLB	Dementia with Lewy Bodies
MSA	Multiple System Atrophy
LBs	Lewy Bodies
LNs	Lewy Neurites
RBD	Rem Behavior Disorder
